# Radiochemical and biological assessments of a PSMA-I&S cold kit for fast and inexpensive ^99m^Tc-labeling for SPECT imaging and radioguided surgery in prostate cancer

**DOI:** 10.3389/fchem.2023.1271176

**Published:** 2023-10-12

**Authors:** Leonardo Lima Fuscaldi, Danielle Vieira Sobral, Ana Claudia Ranucci Durante, Fernanda Ferreira Mendonça, Ana Cláudia Camargo Miranda, Carla Salgueiro, Silvia Gomez de Castiglia, Lilian Yuri Itaya Yamaga, Marcelo Livorsi da Cunha, Luciana Malavolta, Marycel Figols de Barboza, Jorge Mejia

**Affiliations:** ^1^ Hospital Israelita Albert Einstein, Sao Paulo, Brazil; ^2^ Department of Physiological Sciences, Santa Casa de Sao Paulo School of Medical Sciences, Sao Paulo, Brazil; ^3^ Departamento de Química, Universidad Kennedy, Buenos Aires, Argentina; ^4^ Tecnonuclear-Eckert Ziegler, Buenos Aires, Argentina

**Keywords:** PSMA-I&S, technetium-99m, cold kits for radiopharmaceuticals, SPECT imaging, radioguided surgery, prostate cancer

## Abstract

The expression of prostate-specific membrane antigen (PSMA) is upregulated in prostate cancer (PCa) cells and PSMA-ligands have been radiolabeled and used as radiopharmaceuticals for targeted radionuclide therapy (TRT), single photon emission computed tomography (SPECT) or positron emission tomography (PET) molecular imaging, and radioguided surgery in PCa patients. Herein, we aimed at radiolabeling the PSMA-I&S cold kit with ^99m^Tc, resulting in a radiopharmaceutical with high radiochemical yield (RCY) and stability for SPECT imaging and radioguided surgery in PCa malignancies. Various pre-clinical assays were conducted to evaluate the [^99m^Tc]Tc-PSMA-I&S obtained by the cold kit. These assays included assessments of RCY, radiochemical stability in saline, lipophilicity, serum protein binding (SPB), affinity for LNCaP-PCa cells (binding and internalization studies), and *ex vivo* biodistribution profile in naive and LNCaP-PCa-bearing mice. The radiopharmaceutical was obtained with good RCY (92.05% ± 2.20%) and remained stable for 6 h. The lipophilicity was determined to be −2.41 ± 0.06, while the SPB was ∼97%. The binding percentages to LNCaP cells were 9.41% ± 0.57% (1 h) and 10.45% ± 0.45% (4 h), with 63.12 ± 0.93 (1 h) and 65.72% ± 1.28% (4 h) of the bound material being internalized. Blocking assays, employing an excess of unlabeled PSMA-I&S, resulted in a reduction in the binding percentage by 2.6 times. The *ex vivo* biodistribution profile confirmed high accumulation of [^99m^Tc]Tc-PSMA-I&S in the tumor and the tumor-to-contralateral muscle ratio was ∼6.5. In conclusion, [^99m^Tc]Tc-PSMA-I&S was successfully obtained by radiolabeling the cold kit using freshly eluted [^99m^Tc]NaTcO_4_, exhibiting good RCY and radiochemical stability. The preclinical assays demonstrated that the radiopharmaceutical shows favorable characteristics for SPECT imaging and radioguided surgery in PCa patients.

## 1 Introduction

Cancer continues to be a leading source of disability and death worldwide. In 2020, according to the Global Cancer Observatory, there were 10.1 million new cases of cancer and 5.5 million cancer-related deaths among men. For this population, prostate cancer (PCa) corresponds to approximately 1.4 million new cases (14.1%) and 374 thousand cancer-related deaths (6.8%), ranking as the second most prevalent form of cancer. (Ferlay et al., 2021; Bergengren et al., 2023).

The prostate-specific membrane antigen (PSMA) is physiologically expressed in the prostate, kidneys, spleen, small intestine, and lacrimal and salivary glands. (Sheikhbahaei et al., 2017; Fendler et al., 2023). However, it is largely upregulated from 100 to 1,000 times in most PCa tissues, including tumor positive lymph node and bone metastases. (Ghosh and Heston, 2004; Maurer et al., 2016). The recognition of the PSMA as an appropriate target for the PCa lesions opened the opportunity for the development of an extensive array of radiopharmaceuticals intended for theranostic purposes, using targeted radionuclide therapy (TRT) and molecular imaging techniques, i.e., single photon emission computed tomography (SPECT) or positron emission tomography (PET). (Maurer et al., 2016; Arsenault et al., 2018).

In this context, small-molecule PSMA-inhibitors have been developed and labeled with gamma- (^123^I, ^111^In, ^99m^Tc) or positron-emitting radioisotopes (^68^Ga, ^18^F), which allows for the use of SPECT and PET imaging, respectively, for PCa diagnosis and staging. (Afshar-Oromieh et al., 2014; Robu et al., 2017; Fuscaldi et al., 2021). Almost simultaneously, small-molecule PSMA-inhibitors have also been labeled with beta- (^177^Lu, ^90^Y) or alpha-emitting radioisotopes (^225^Ac), to be used as TRT agents, in this way working as theranostic pairs. (Ruigrok et al., 2019). Until now, the most successful PSMA-inhibitor, which presents great specificity and affinity for the PSMA active center, is the glutamate–urea–lysine inhibitor. This urea-based PSMA-inhibitor has been modified with different chelators through different chemical linkers for labeling with different radioisotopes. (Afshar-Oromieh et al., 2014; Maurer et al., 2016). Among their analogues, the ^68^Ga-labeled PSMA-11 was the first PET-radiopharmaceutical for targeting PSMA-upregulated lesions in men with PCa and the only metal-based radiopharmaceutical including a small-molecule PSMA-inhibitor pharmacophore approved by the FDA-USA for patients with PCa and suspected biochemical recurrence. (Carlucci et al., 2021). Another possibility for the small-molecule PSMA-inhibitors is their use as radioguided surgery agents.

Radioguided surgery for selective lymphadenectomy has been used for more than 2 decades, mainly in melanoma and breast cancer, following the concept of identifying the sentinel lymph node. (Povoski et al., 2009; Moncayo et al., 2015). In PCa, initial evidence is beginning to emerge, primarily related to salvage lymphadenectomy in cases with one or a few involved lymph nodes and an absence of distant lesions. (Ploussard et al., 2019). For that, urea-based PSMA-inhibitors, such as the ^111^In-labeled analogue [^111^In]In-PSMA-I&T, have been successfully employed. [^111^In]In-PSMA-I&T radioguided surgery has demonstrated improved intraoperative identification and resection of metastatic PSMA-positive lymph nodes. (Maurer et al., 2015; Schottelius et al., 2015). However, its high cost, reduced availability, and great radiation exposure hamper its broader clinical application. (Robu et al., 2017). On the other hand, ^99m^Tc is promptly available from a ^99^Mo/^99m^Tc generator, with low cost. Additionally, its low gamma energy (∼141 keV) and half-life (∼6 h) make it more suitable for gamma-cameras and radioguided surgery. (Fuscaldi et al., 2014; Carlesso et al., 2015). Consequently, the development of a^99m^Tc-labeled PSMA-inhibitor analogue appears to hold great promise for both SPECT imaging and radioguided surgery in PCa.

Another urea-based PSMA inhibitor analogue, PSMA-I&S (Robu et al., 2017), when radiolabeled with ^99m^Tc, has been used in the intrasurgery identification of metastatic lymph nodes, with confirmed applicability in radioguided surgery of PCa and potential to be also used as a SPECT imaging alternative to the PET-based radiopharmaceuticals. Initial studies have confirmed the applicability of [^99m^Tc]Tc-PSMA-I&S in radioguided surgery of PCa. (Kratzik et al., 2018; Maurer et al., 2019).

**FIGURE 1:**
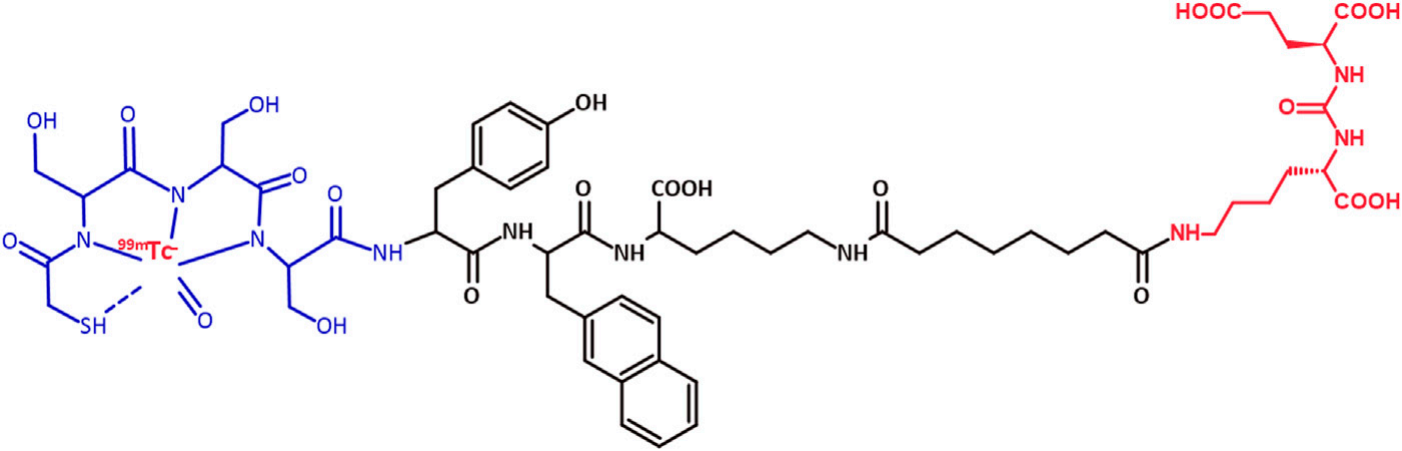
Chemical structure of [^99m^Tc]Tc-PSMA-I&S. Red: glutamate–urea–lysine PSMA inhibitor and ^99m^Tc; Black: spacer; Blue: NHS-*S*-acetylmercaptoacetyltriserine (NHS-MAS_3_) chelator.

Herein, we aimed at assessing a PSMA-I&S cold kit that would allow for fast, simple, and efficient ^99m^Tc-labeling by simply adding the fresh eluate of a regular ^99^Mo/^99m^Tc generator to the vial containing the freeze-dried reagent. We evaluated radiochemical and biological parameters of this new PSMA-I&S cold kit, which could potentially be used in hospital settings similarly to other ^99m^Tc-radiopharmaceuticals, routinely generating [^99m^Tc]Tc-PSMA-I&S (Figure 1) in Nuclear Medicine services. We also report our first human application of [^99m^Tc]Tc-PSMA-I&S, obtained from this cold kit, in a patient with PCa who underwent radioguided surgery for the identification and subsequent resection of both the primary prostate lesion and metastatic lymph nodes.

## 2 Materials and methods

### 2.1 Radiolabeling of the PSMA-I&S with ^99m^Tc

The PSMA-I&S cold kit was produced and supplied by *Tecnonuclear-Eckert Ziegler* (Argentina). The formulation of this cold kit had been previously optimized, and the final composition includes: 80 µg of PSMA-I&S, 5 mg of sodium tartrate, 30 µg of ascorbic acid, 20 mg of mannitol, and 25 µg of stannous chloride. This formulation resulted in a sterile and apyrogenic lyophilized reagent with a shelf stability of 7 months when stored under refrigeration (4–8 °C) conditions (unpublished data). For the radiolabeling procedure, ^99m^Tc was eluted from a ^99^Mo/^99m^Tc generator (IPEN-TEC 88800 MBq—*Instituto de Pesquisas Energéticas e Nucleares*, Brazil) in saline yielding a [^99m^Tc]NaTcO_4_ solution. A 1.5 mL aliquot of freshly eluted [^99m^Tc]NaTcO_4_ solution (814–925 MBq) was directly added to the flask containing the freeze-dried reagent and the solution was heated (100 °C) for 20 min. The evaluation of radiochemical yield (RCY) involved two types of chromatographic analyses: ascending chromatography and reversed-phase high-performance liquid chromatography (RP-HPLC).

In the ascending chromatography, a dual system was employed using an instant thin layer chromatographic glass microfiber impregnated with silica gel (ITLC-SG) strip (Merck, Germany) with a 1 M NH_4_OAc solution:MeOH (1:1) as mobile phase and a reversed phase-18 modified silica gel thin layer chromatographic (TLC-RP18) strip (Merck, Germany) with 1% trifluoroacetic acid (TFA) in acetonitrile:water (ACN:H_2_O- 30:70) as mobile phase. Subsequently, a radio-TLC imaging scanner (AR-2000 — Eckert and Ziegler, Germany) was used to determine the radioactivity distribution on the strips. The retention factors (R_f_) of [^99m^Tc]NaTcO_4_, [^99m^Tc]TcO_2_, and [^99m^Tc]Tc-PSMA-I&S were determined, along with the percentage of radioactivity around their respective peaks.

For the RP-HPLC analyses, an ultra HPLC system (1,290 Infinity II UHPLC - Agilent Technologies, United States), equipped with a radioactivity detector (Flow-count base B-FC-1000 Model 106 — Eckert and Ziegler, Germany), was utilized. An analytical C18 column (150 mm × 4.6 mm; 5 µm) was utilized, maintained at 25 °C, with mobile phases A (0.1% TFA/H_2_O) and B (0.1% TFA/ACN). The mobile phase B underwent a gradient elution with percentages ranging from 5% to 10% (0–2 min), 10%–30% (2–16 min), and 30%–5% (16–22 min); flow rate = 1 mL min^-1^. The UV (*λ* = 284 nm) and radioactivity detectors were used to obtain the signals. The retention times (R_t_) of the unlabeled precursor PSMA-I&S, [^99m^Tc]NaTcO_4_, and [^99m^Tc]Tc-PSMA-I&S were determined.

### 2.2 Radiochemical stability assessment

The radiochemical stability was assessed as previously reported. (Fuscaldi et al., 2021). The assessment was conducted in saline (room temperature) from immediate to 6 h after the radiolabeling process (*n* = 3). At each specified time point during this interval, samples of [^99m^Tc]Tc-PSMA-I&S were subjected to both chromatographic analyses, described in Section 2.1.

### 2.3 Partition coefficient determination

The partition coefficient (P) was determined in a mixture containing 500 µL of *n*-octanol, 450 µL of water, and 50 µL of [^99m^Tc]Tc-PSMA-I&S in 0.9% saline solution (∼30 MBq; 14.1 MBq/nmol) - (*n* = 6), following the procedure previously described. (Durante et al., 2019). After complete separation of the organic and aqueous phases through centrifugation, 100 µL aliquots from each phase were measured using an automated gamma counter (Wizard2™ 3” 2,480 — PerkinElmer, United States). The lipophilicity, expressed as Log P, was calculated as the logarithm of the ratio between the radioactivity counts in the organic and aqueous phases, which provided an estimation of the extent to which [^99m^Tc]Tc-PSMA-I&S distributes to the organic and the aqueous phases.

### 2.4 Serum protein binding evaluation

The evaluation of serum protein binding (SPB) was conducted using Amicon^®^ ultra centrifugal filters (10 kDa molecular weight cut-off [MWCO]). A 50 µL aliquot of [^99m^Tc]Tc-PSMA-I&S in saline solution (∼30 MBq; 14.1 MBq/nmol) was incubated with 450 µL of fresh mouse serum (37 °C) for 60 min (*n* = 6). After the incubation period, the serum proteins were separated from the solution using the centrifugal filters through a centrifugation step. The pellets (representing the fraction bound to the serum proteins and retained by the filter) and supernatants (representing the fraction collected after centrifugation) were measured using an automated gamma counter (Wizard2™ 3” 2,480 — PerkinElmer, United States). The SPB was determined by calculating the ratio between the radioactivity counts in the pellet and the total radioactivity counts (pellet + supernatant), which provided an estimation of the extent to which [^99m^Tc]Tc-PSMA-I&S binds to serum proteins.

### 2.5 LNCaP cells culture

LNCaP human cells were cultivated according to the commercial supplier`s recommendations: RPMI-1640 medium enriched with 2 mM L-glutamine, 1 mM sodium pyruvate, 1% v/v antibiotics (penicillin and streptomycin), and 10% v/v fetal bovine serum; incubation in a humidified 5% CO_2_ atmosphere (37 °C). When ready for use, the cells were re-suspended either in enriched RPMI-1640 medium (*in vitro* assays) or a matrigel:enriched RPMI-1640 medium (1:1) mixture (prostate tumor animal model establishment).

### 2.6 *In vitro* affinity for LNCaP cells

The *in vitro* affinity was assessed as the binding and internalization percentages to LNCaP cells following previously established methods. (Fuscaldi et al., 2015). In brief, a 50 µL aliquot of [^99m^Tc]Tc-PSMA-I&S in saline solution (∼30 MBq; 14.1 MBq/nmol) was incubated with 2 × 10^6^ non-adhered LNCaP cells re-suspended in 450 µL of enriched RPMI-1640 medium (37 °C) for 1 and 4 h (*n* = 5 for each time point). After centrifugation, the resulting cell pellets (representing the fraction bound to the cells) and supernatants (representing the fraction collected after centrifugation) were measured using an automated gamma counter (Wizard2™ 3″ 2,480 — PerkinElmer, United States). The binding percentage was calculated as the ratio between the radioactivity counts in the pellet and the total radioactivity counts (pellet + supernatant), which provided an estimation of the extent to which [^99m^Tc]Tc-PSMA-I&S binds to LNCaP cells. Subsequently, a 500 µL aliquot of 0.2 M acetic acid in 0.5 M NaCl solution (pH = 2.8) was added to the cell pellets for 5 min to remove [^99m^Tc]Tc-PSMA-I&S bound to the cell surface. Again, post-centrifugation, the resulting cell pellets (now representing the fraction internalized in the cells among the bound fraction) and supernatants (representing the fraction collected after centrifugation) were measured using an automated gamma counter (Wizard2™ 3” 2,480 — PerkinElmer, United States). The internalization percentage was calculated similarly to the binding percentage, providing an estimation of the extent to which [^99m^Tc]Tc-PSMA-I&S internalizes to LNCaP cells.

### 2.7 *In vitro* specific affinity for LNCaP cells

The *in vitro* specific affinity was conducted in 12-well adherent plate and assessed as the binding percentages to unblocked and blocked LNCaP cells, following previously established protocols. (Sobral et al., 2022). In brief, 2 × 10^5^ LNCaP cells re-suspended in 500 µL of enriched RPMI-1640 medium were seeded onto each well of the 12-well adherent plate and allowed to adhere for 24 h. For the unblocked specific binding study, the medium was removed and a 50 µL aliquot of [^99m^Tc]Tc-PSMA-I&S in saline solution (∼30 MBq; 14.1 MBq/nmol) was mixed to a 450 µL aliquot of enriched RPMI-1640 medium and added to the wells (*n* = 5), while for the blocked non-specific binding study, an excess of 500× of the corresponding unlabeled PSMA-I&S (competitor) was added 15 min prior to the radiolabeled compound (*n* = 5). In both cases, the plates were incubated in a humidified 5% CO_2_ atmosphere (37 °C) for 4 h. Subsequently, for each well, the supernatant was removed and the well washed with PBS, which was combined with the respective supernatant. The cells were then trypsinized and transferred to microtubes, and each well was washed again with PBS and combined with the respective trypsinized cells. The pellets (representing the fraction bound to the cells) and supernatants (representing the fraction not bound to the cells) were measured using an automated gamma counter (Wizard2™ 3” 2,480 — PerkinElmer, United States). The binding percentage was calculated as described in section 2.6.

### 2.8 LNCaP prostate tumor animal model

Male BALB/c nude mice (∼8 weeks old; ∼20 g) received unrestricted access to water and food, and were housed in specific pathogen-free conditions. The housing facility maintained a controlled environment with a temperature of 22°C ± 2 °C, humidity of 50% ± 10%, and a regulated light-dark cycle of 12:12 h. All animal procedures were approved by the Ethics Committee on Animal Use of our institution, under the protocol number 4005/19, adhering to the Brazilian Guidelines on Care and Use of Animals for Scientific Research or Teaching Activities.

To establish the tumor model, a 200 µL aliquot containing 1 × 10^7^ LNCaP cells in matrigel:enriched RPMI-1640 medium (1:1) mixture was subcutaneously inoculated into the right upper flank of the animals, following established methods with some modifications. (Fuscaldi et al., 2015). The tumor development was accompanied for a period <40 days (diameter ∼10 mm). The [^99m^Tc]Tc-PSMA-I&S *ex vivo* biodistribution profile was obtained in this PCa animal model.

### 2.9 [^99m^Tc]Tc-PSMA-I&S *ex vivo* biodistribution profile

A total of 12 male BALB/c nude mice were included in the [^99m^Tc]Tc-PSMA-I&S *ex vivo* biodistribution study, with 6 mice having LNCaP tumors (LNCaP-tumor-bearing group) and 6 mice being naive (control group). The mice were anesthetized by intraperitoneal administration of ketamine and xylazine combination (100:10 mg/kg), and a 100 µL aliquot of the radiopharmaceutical (∼7.4 MBq; 14.1 MBq/nmol) combined with saline solution was intravenously injected. After 1 h from the injection, the mice were euthanized, and specific organs and tissues were removed from the animals. The weight of each organ or tissue was recorded, and the corresponding radioactivity was measured using an automated gamma counter (Wizard2™ 3” 2,480 — PerkinElmer, United States), along with a ^99m^Tc standard dose with the same radioactivity amount as injected into the mice and defined as 100%. The percentage of injected dose per gram of tissue (%ID/g) for each organ or tissue was calculated, enabling the evaluation of [^99m^Tc]Tc-PSMA-I&S *ex vivo* biodistribution profile.

### 2.10 Clinical case

A 59-year-old patient diagnosed with PCa underwent a [^68^Ga]Ga-PSMA-11 PET/CT for staging. Twenty-one days later, the same patient underwent a [^99m^Tc]Tc-PSMA-I&S SPECT/CT and subsequent radioguided surgery to resect the primary tumor and the lymph node metastases. The patient received an intravenous administration of [^99m^Tc]Tc-PSMA-I&S (592 MBq; 14.1 MBq/nmol), obtained from the cold kit, 14 h prior to an open radical prostatectomy and extended pelvic lymphadenectomy. [^99m^Tc]Tc-PSMA-I&S SPECT/CT images were acquired 2 h after the administration of the radiopharmaceutical. The pilot study received approval from the institutional ethics committee and the patient signed the corresponding informed consent to use the SPECT images for research purposes.

### 2.11 Statistical analysis

Quantitative data were presented as either “mean ± standard deviation (SD)" for *in vitro* data or “mean ± standard error of the mean (SEM)" for *in vivo* data. The comparison of paired means was conducted using the Student’s t-test, while for comparisons involving three or more groups, Analysis of Variance (ANOVA) followed by Tukey’s multiple comparisons test was employed (*p*-value = 0.05).

## 3 Results

**FIGURE 2:**
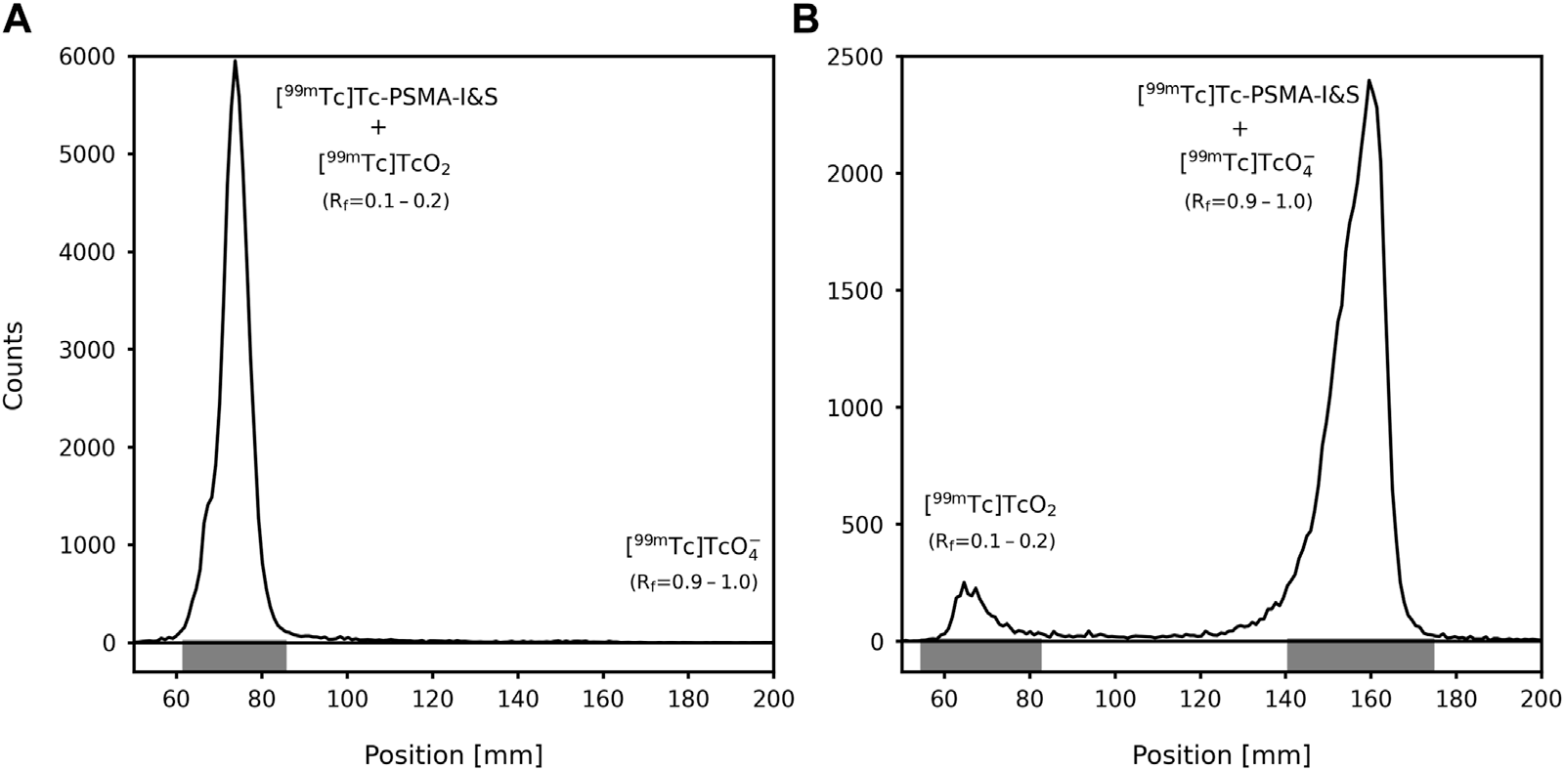
Radiochromatograms obtained using an ascending chromatographic double system: **(A)** TLC-RP18 strip with 1% TFA in ACN:H_2_O (3:7) as the eluent; **(B)** ITLC-SG strip with 1 M AcONH_4_:MeOH (1:1) as the eluent. R_f_: Retention factor.

**FIGURE 3:**
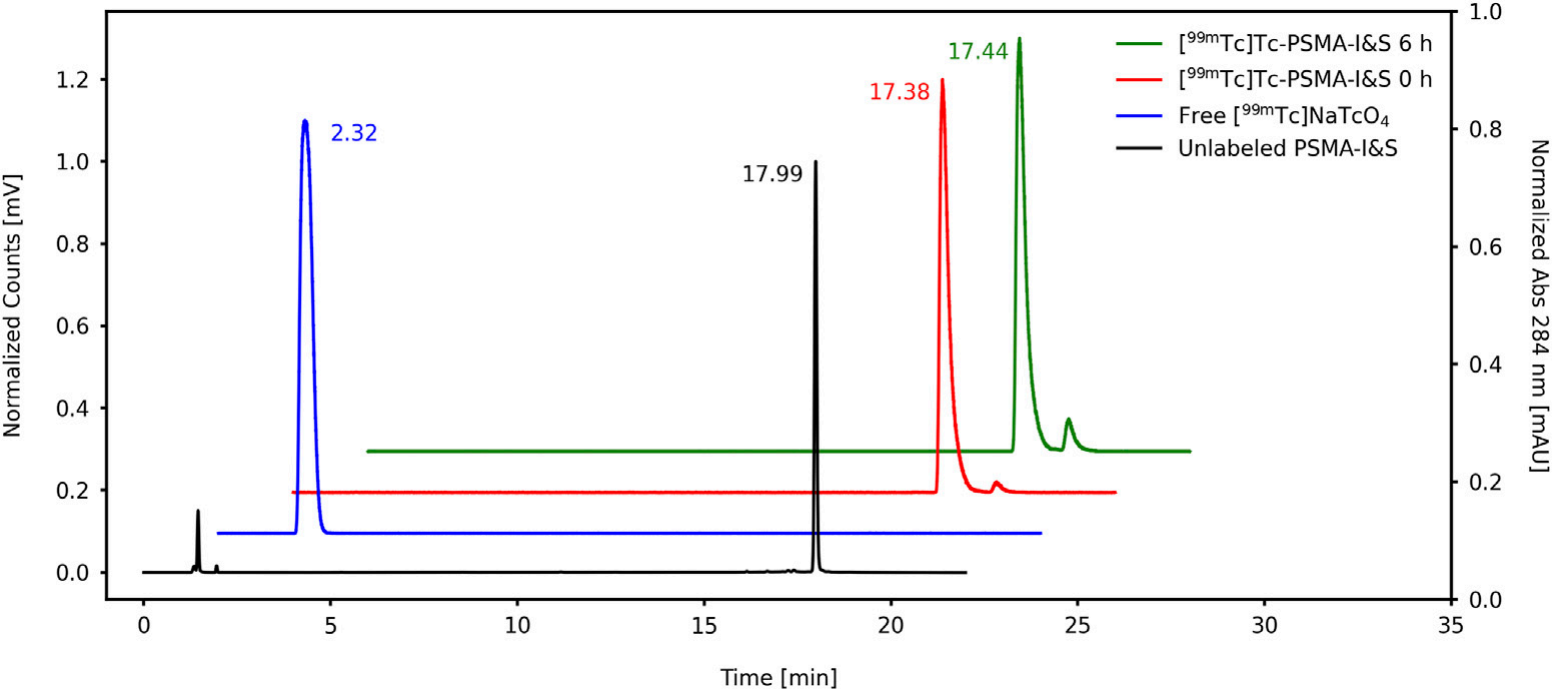
RP-HPLC chromatograms of the unlabeled PSMA-I&S, free [^99m^Tc]‐NaTcO_4_, and [^99m^Tc]Tc-PSMA-I&S (immediate and radiochemical stability at 6 h).

The radiolabeling of the PSMA-I&S cold kit was performed using freshly eluted [^99m^Tc]NaTcO_4_ in saline, obtaining RCY of 92.05% ± 2.20% (*n* = 5) and neutral final solution (pH = 6.5–7.0). In the ascending chromatographic dual system, used to evaluate the RCY, both mobile phases dragged the [^99m^Tc]TcO_4_^−^ to the elution front (R_f_ = 0.9–1.0). Conversely, [^99m^Tc]TcO_2_ remained at the point of application on the strips (R_f_ = 0.1–0.2) also when both mobile phases were used. [^99m^Tc]Tc-PSMA-I&S migrates to the top of the ITLC-SG strip when 1 M AcONH_4_:MeOH (1:1) was used as the eluent (R_f_ = 0.9–1.0) and remains at the point of application of the TLC-RP18 strip when 1% TFA in ACN:H_2_O (3:7) was used as the eluent (R_f_ = 0.1–0.2)—(Figure 2). This result agrees with the data provided by RP-HPLC analyzes (Figure 3). Both chromatographic analyzes were also used to assess the [^99m^Tc]Tc-PSMA-I&S radiochemical stability in saline (room temperature) over a period of 6 h. The ascending chromatographic data (Table 1) indicate that [^99m^Tc]Tc-PSMA-I&S remained stable in saline during the entire evaluation time (*p*-value = 0.7496; *n* = 3). The RP-HPLC results confirmed the findings from the ascending chromatographic data, and Figure 3 shows a radiochromatogram of [^99m^Tc]Tc-PSMA-I&S at 6 h post-radiolabeling process.

**TABLE 1:** Radiochemical stability of [^99m^Tc]Tc-PSMA-I&S in saline (room temperature), assessed through dual system ascending chromatography.

Time [h]	RCP [%]
0	92.05 ± 2.20
1	89.20 ± 4.50
2	89.10 ± 5.94
4	88.25 ± 5.84
6	88.89 ± 3.26

The values are expressed as “mean ± SD”: (*n* = 3). No statistically significant difference was observed within the time interval (*p* = 0.7496). RCP: radiochemical purity.

The log P value for [^99m^Tc]Tc-PSMA-I&S was determined to be −2.41 ± 0.06 (*n* = 6), confirming its hydrophilic characteristics. Additionally, the SPB of [^99m^Tc]Tc-PSMA-I&S was 97.67% ± 0.30% (*n* = 6).

**FIGURE 4:**
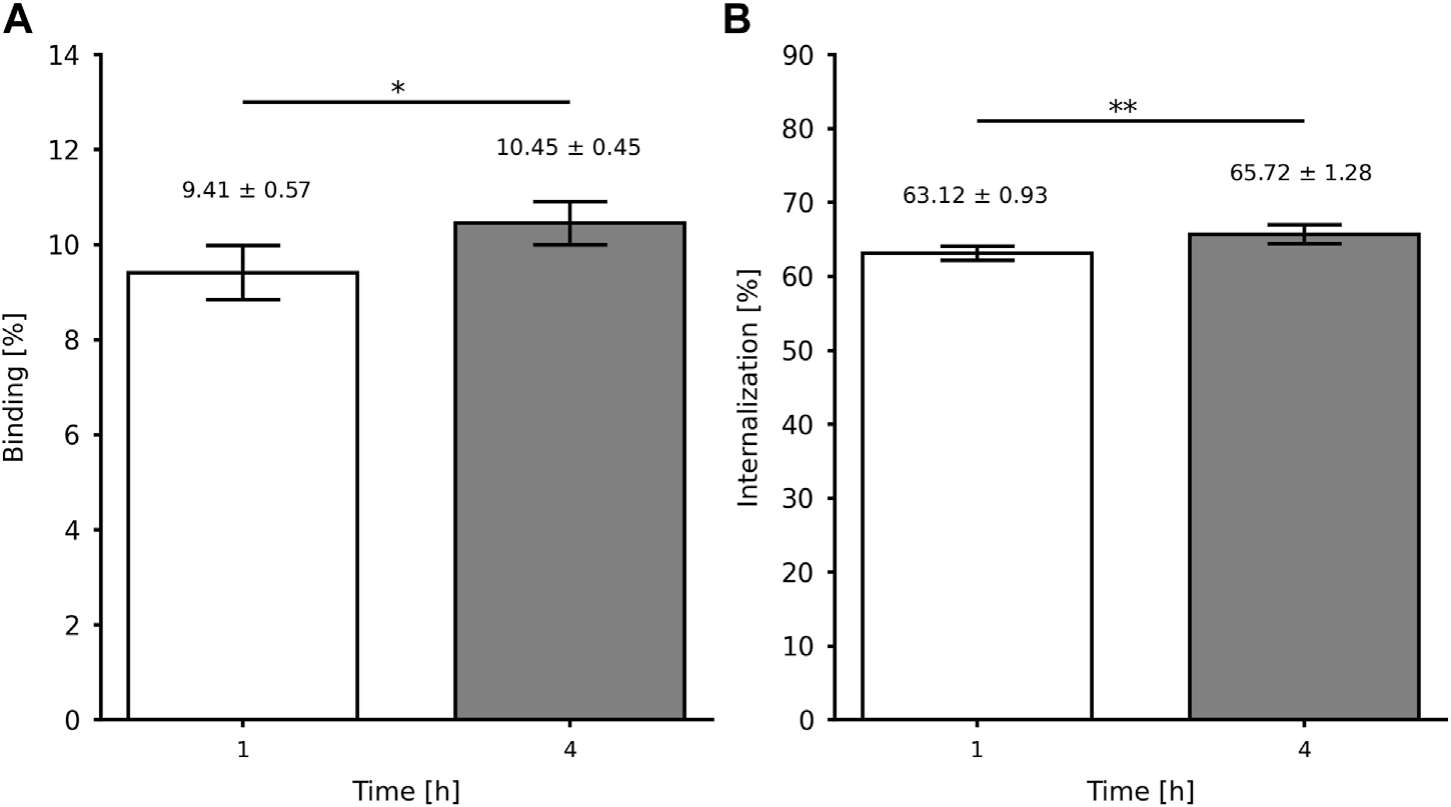
*In vitro* affinity of [^99m^Tc]Tc-PSMA-I&S for LNCaP cells (2 × 10^6^): **(A)** binding and **(B)** internalization percentages. Data are expressed as “mean ± SD” (*n* = 5). *p-value < 0.05 and **p-value < 0.01.

**FIGURE 5:**
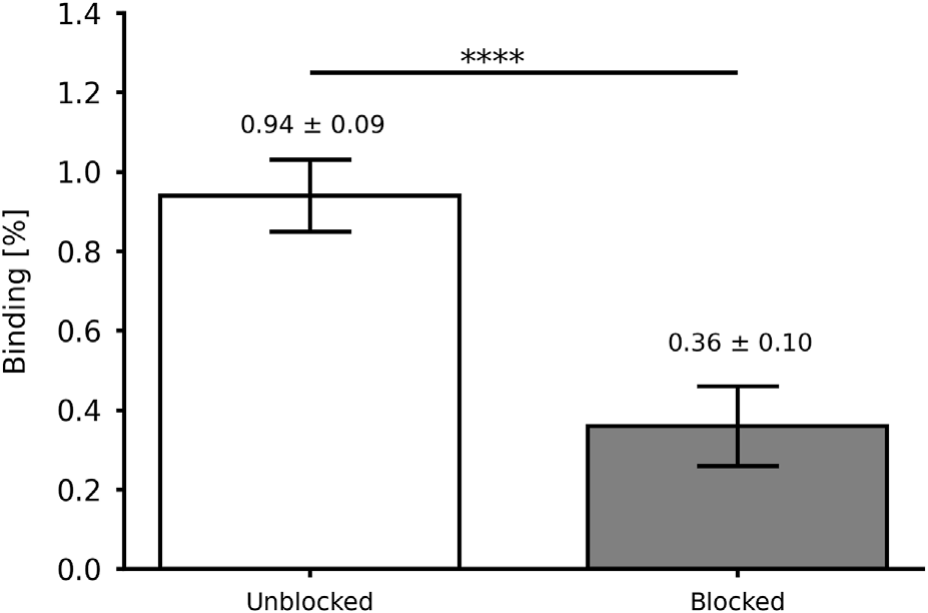
*In vitro* specific affinity of [^99m^Tc]Tc-PSMA-I&S for LNCaP cells (2 × 10^5^) at 4 h after incubation. Data are expressed as “mean ± SD” (*n* = 5). ****p-value < 0.001.

Figure 4 summarizes the *in vitro* affinity data, showing a significant increment in the binding percentage from 9.4% to 10.5% (*p*-value = 0.0135; *n* = 5) and the internalization percentage from 63.1% to 65.7% (*p*-value = 0.0063; *n* = 5) at 1 and 4 h, respectively. Furthermore, the *in vitro* specific affinity (Figure 5) exhibited a decrease of ∼2.6 times in the binding percentage from the unblocked to the blocked group (*p*-value < 0.0001; *n* = 5).

**FIGURE 6:**
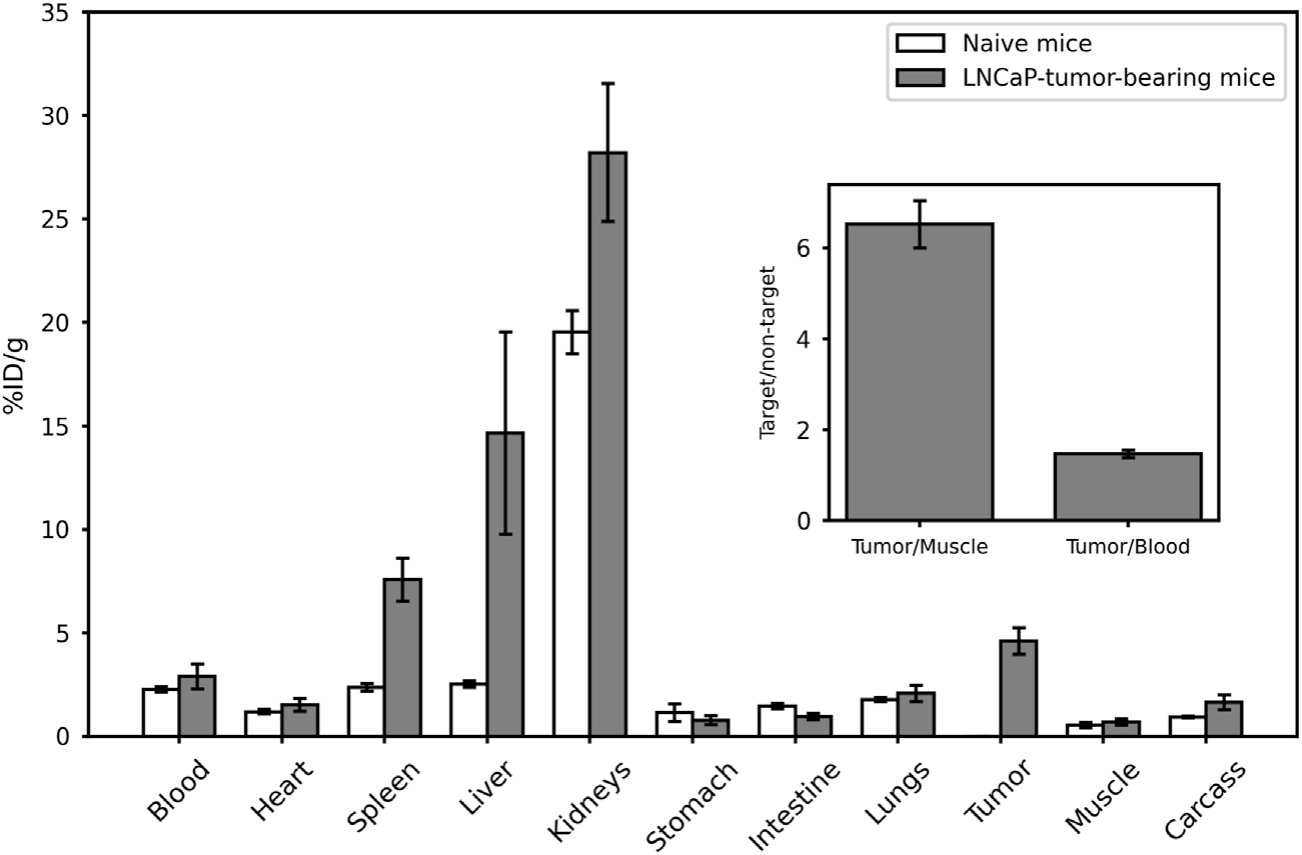
[^99m^Tc]Tc-PSMA-I&S *ex vivo* biodistribution profile (60 min). The insert within the figure shows the target-to-non-target ratios, calculated based on the *ex vivo* biodistribution data from LNCaP-tumor-bearing mice. Data are represented as “mean ± SEM” (*n* = 6).

Finally, the [^99m^Tc]Tc-PSMA-I&S *ex vivo* biodistribution profile (Figure 6) was obtained following intravenous injection in both naive and LNCaP-tumor-bearing mice. The results revealed significant accumulation in the kidneys, likely due to urinary excretion, and the spleen, which is known for its physiological PSMA expression. For the xenografted LNCaP-tumor-bearing mice, the *ex vivo* biodistribution data revealed approximately 5% uptake of [^99m^Tc]Tc-PSMA-I&S by the tumor. Moreover, the calculated tumor-to-blood and tumor-to-contralateral muscle ratios were approximately 1.5 and 6.5, respectively (Figure 6—insert). These findings indicate a preferential accumulation of the radiopharmaceutical in the tumor site compared to surrounding muscle and blood tissues.

**FIGURE 7:**
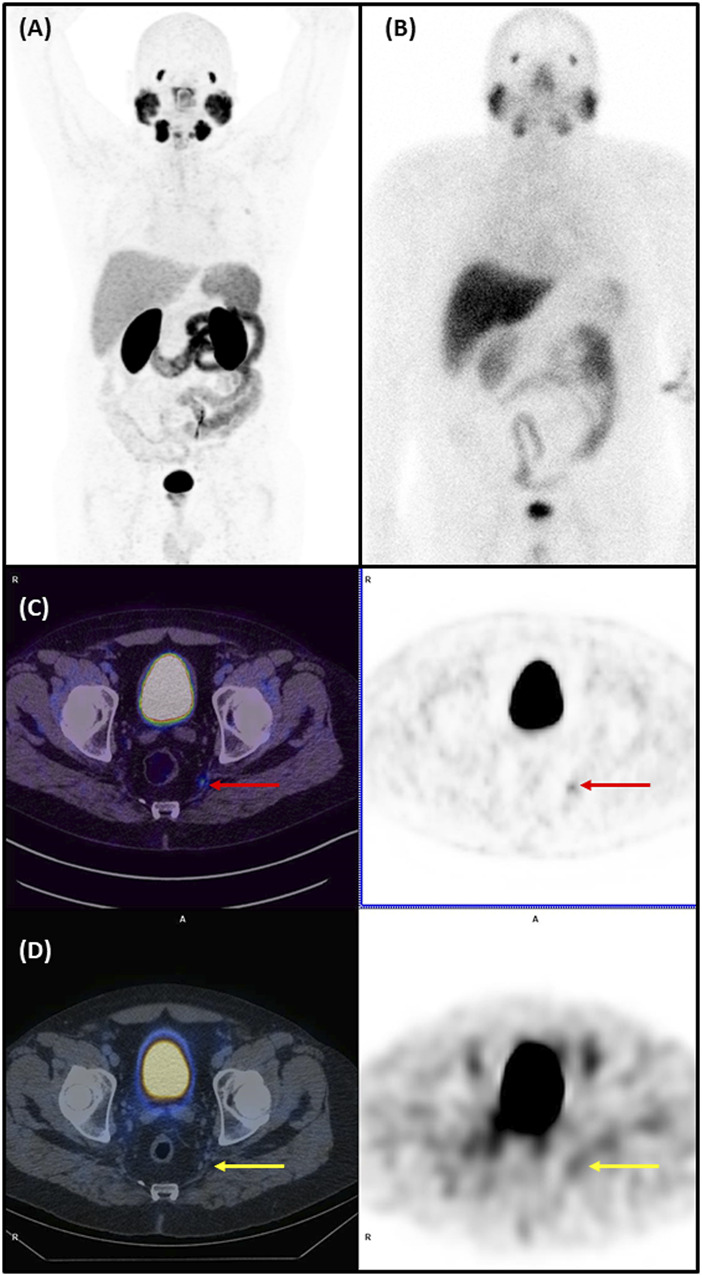
[^68^Ga]Ga-PSMA-11 PET/CT and [^99m^Tc]Tc-PSMA-I&S SPECT/CT of a 59-year-old man with a Gleason score of 7 (4 + 3) prostate cancer (PCa). **(A)** The maximum intensity projection (MIP) of [^68^Ga]Ga-PSMA-11 PET/CT imaging (anterior view) performed for staging. **(B)** [^99m^Tc]Tc-PSMA-I&S whole body scan (anterior view), obtained the day before radioguided surgery. **(C)** [^68^Ga]Ga-PSMA-11 PET/CT of the pelvic region: *Left* - fused PET/CT axial slice of the pelvis showing abnormal uptake in a small (8 mm) left internal iliac lymph node (red arrow); *Right* - corresponding PET axial slice, pinpointing abnormal [^68^Ga]Ga-PSMA-11 uptake in the above mentioned lymph node. **(D)** [^99m^Tc]Tc-PSMA-I&S SPECT/CT of the pelvic region: *Left*–fused SPECT/CT axial slice of the pelvis showing slight abnormal uptake in the same small (8 mm) left internal iliac lymph node (yellow arrow); *Right* - corresponding SPECT axial slice, pinpointing abnormal [^99m^Tc]Tc-PSMA-I&S uptake in the earlier quoted lymph node. _***(Nuclear Medicine Department - Hospital Israelita Albert Einstein, Brazil)***_

The clinical application of the [^99m^Tc]Tc-PSMA-I&S obtained from the evaluated PSMA-I&S cold kit was demonstrated in a 59-year-old PCa patient (ISUP grade = 3; PSA level = 25.7 ng/mL), exhibiting no evidence of metastatic disease during preoperative staging, which included bone scintigraphy and pelvic magnetic resonance imaging. However, [^68^Ga]Ga-PSMA-11 PET/CT (Figures 7A,C) revealed radiopharmaceutical uptake in the primary prostatic lesion and a suspicious left internal iliac lymph node measuring 0.8 cm, suggestive of lymph node metastasis. Subsequent [^99m^Tc]Tc-PSMA-I&S SPECT/CT imaging (Figures 7B,D), conducted 2 h after radiopharmaceutical administration, confirmed the presence of a PSMA-avid primary prostate tumor and left internal iliac lymph node metastasis, in concordance with the findings from the [^68^Ga]Ga-PSMA-11 PET/CT scan. During surgery, the surgeons successfully identified and resected the radioactive lymph node specimen using a gamma probe. Histopathological analysis of the resected lymph node confirmed metastatic disease.

## 4 Discussion

The ^99m^Tc-radiolabeling process generates two main radiochemical impurities, [^99m^Tc]TcO_4_^−^ and [^99m^Tc]TcO_2_. These impurities tend to accumulate in specific organs, with [^99m^Tc]TcO_4_^−^ accumulating in the thyroid and stomach, while [^99m^Tc]TcO_2_ in the liver and spleen. (Fuscaldi et al., 2014). For clinical applications, it is recommended that ^99m^Tc-labeled radiopharmaceuticals have low levels of radiochemical impurities, typically below 10%, to maintain their efficacy. High radiochemical impurities can lead to undesirable biodistribution and hamper imaging quality. The evaluated cold kit generated [^99m^Tc]Tc-PSMA-I&S with RCY of ∼92%. In the ascending chromatography, the ITLC-SG strip/1 M NH_4_OAc solution:MeOH (1:1) system was used to determine the amount of [^99m^Tc]TcO_2_, whereas the TLC-RP18 strip/1% TFA in ACN:H_2_O (3:7) system was used to quantify [^99m^Tc]TcO_4_^−^. In the RP-HPLC analyses, it is important to note that colloidal [^99m^Tc]TcO_2_ binds to the C18 column and does not elute with the solvent gradient. On the contrary, free [^99m^Tc]TcO_4_^−^ does not exhibit binding to the C18 column and elutes early in the radiochromatogram profile (R_t_ = 2.32 min). Therefore, RP-HPLC analyses serve as a reliable method for confirming the presence or absence of free [^99m^Tc]TcO_4_^−^ in the final product. However, it should be acknowledged that RP-HPLC lacks accuracy when it comes to assessing [^99m^Tc]TcO_2_ levels. Furthermore, the ascending chromatography is faster, cost-effective, and more accessible than the RP-HPLC analyses for implementation in a radiopharmacy setting. The radiolabeling procedure of the cold kit yielded almost no free [^99m^Tc]TcO_4_^−^. However, the main impurity present in the final solution was colloidal [^99m^Tc]TcO_2_. According to local legislation, when a RCY exceeds 90%, no further purification steps are conducted. If a RCY below 90% is obtained, the solution can be purified using a C18 cartridge. Besides, the [^99m^Tc]Tc-PSMA-I&S presented high radiochemical stability in saline over a period of 6 h. Thus, the radiolabeling of the PSMA-I&S cold kit with ^99m^Tc is able to produce [^99m^Tc]Tc-PSMA-I&S with a RCY of ∼90% and good radiochemical stability, allowing for appropriate clinical application.

The Log P value was calculated based on the P between *n*-octanol and water. A negative Log P value (−2.4) indicates that [^99m^Tc]Tc-PSMA-I&S is hydrophilic, which is important to consider when evaluating its behavior and biodistribution in biological systems. (Silverman and Holladay, 2014). Hydrophilic compounds are typically eliminated through urinary excretion, resulting in rapid blood clearance. This advantage contributes to a better imaging background. Previous research reported a Log P value of −3.0 for [^99m^Tc]Tc-PSMA-I&S obtained through a non-kit-type radiochemistry method. (Robu et al., 2017). Although published data also suggest that the radiopharmaceutical falls within the hydrophilic range, the difference from our result may stem from variations in our final solution`s composition. Furthermore, high SPB values were observed (∼97%), albeit without correction for potential non-specific binding to the filter material. These values were slightly higher than those reported by Robu and co-workers (∼94%), who corrected their data for non-specific binding. (Robu et al., 2017).

The binding of [^99m^Tc]Tc-PSMA-I&S to LNCaP cells encompasses the total amount of molecules that have bound to the cell surface, including the fraction that has been subsequently internalized. This binding process can be attributed to the presence of PSMA on the surface of LNCaP cell membranes, facilitating the interaction with the radiopharmaceutical. (Israeli et al., 1993). On the other hand, internalization refers to the specific fraction of the bound [^99m^Tc]Tc-PSMA-I&S that has been internalized in the LNCaP cells. (Fuscaldi et al., 2015). Both binding and internalization processes are critical factors to consider when evaluating the interaction of a radiopharmaceutical with tumor cells and understanding its potential as a targeted radiopharmaceutical for tumors. The present data are higher than those reported for [^99m^Tc]Tc-EDDA/HYNIC-iPSMA (Ferro-Flores et al., 2017) and [^68^Ga]Ga-PSMA-11 (Fuscaldi et al., 2021), but lower than those reported for the same [^99m^Tc]Tc-PSMA-I&S (Robu et al., 2017). In this case, Robu and co-workers compared the binding of the radiopharmaceutical with a reference compound [(^125^I-BA (KuE)]. Furthermore, in this study, we also evaluated the *in vitro* specific affinity of [^99m^Tc]Tc-PSMA-I&S for LNCaP cells either with (blocked) or without (unblocked) the presence of an excess of the unlabeled PSMA-I&S (competitor). The excess of unlabeled PSMA-I&S was able to block the PSMA binding site, reducing the binding percentage. These data also confirm the specificity of the PSMA-I&S for the PSMA upregulated on the membrane surface of LNCaP cells.

The [^99m^Tc]Tc-PSMA-I&S *ex vivo* biodistribution profile showed elimination through the urinary tract, as evidenced by high uptake in the kidneys. This observation aligns with the hydrophilic feature of the radiopharmaceutical, indicated by its Log P < 0. The significant uptake of [^99m^Tc]Tc-PSMA-I&S by the spleen, although not a specific target, has been reported in animal (Kopka et al., 2017; Fuscaldi et al., 2021) and human studies (Ferreira et al., 2019), and it is attributed to its physiological PSMA expression. (Sheikhbahaei et al., 2017; Fendler et al., 2023). Some variations in the biodistribution profile were observed for certain organs, notably the liver, spleen, and kidneys, when compared to previous data (Robu et al., 2017), which could be due to differences in the pharmacokinetics of the radiopharmaceutical arising from i) variations in the final composition of the cold kit, ii) differences in animal anesthesia protocols, with our study employing ketamine and xylazine, while Robu and coworkers’ study utilized isoflurane, and iii) distinctions in the mouse species, as our work involved nude Balb/c mice, whereas Robu and coworkers employed CB17 severe combined immunodeficiency mice. These factors collectively underscore the importance of considering experimental conditions and animal models when interpreting and comparing biodistribution data across studies. Furthermore, it is worth mentioning that the higher levels of radioactivity accumulation observed in the spleen and liver during the experiments with the tumor group could potentially be attributed to a greater quantity of [^99m^Tc]TcO_2_, considering that our RCY was measured at 92.05% ± 2.20%. However, the primary interest lies in the high [^99m^Tc]Tc-PSMA-I&S uptake by the tumor, which leads to a substantial tumor-to-contralateral muscle ratio equals to 6.5. The reason for this can be linked to the overexpression of PSMA on the membrane of LNCaP cells (Israeli et al., 1993), as confirmed by our *in vitro* assays where the radiopharmaceutical specifically bound to the antigen upregulated in the cells and subsequently was internalized. These findings support the *in vivo* specificity and affinity of [^99m^Tc]Tc-PSMA-I&S for prostate tumor tissue and suggest its suitability for use in SPECT imaging and radioguided surgery with sufficient quality in PCa applications.

The development of a PSMA analogue cold kit for ^99m^Tc labeling is of great significance, given that >85% of Nuclear Medicine procedures utilize ^99m^Tc-labeled radiopharmaceuticals. Additionally, many Nuclear Medicine services rely solely on gamma-cameras, making it desirable to have a substitute for PET radiopharmaceuticals that can enhance PSMA applicability in such centers. Moreover, the production of PET radioisotopes predominantly involves cyclotrons, which are expensive and pose challenges in distribution, limiting their widespread use.

Finally, only a ^99m^Tc-labeled PSMA can be employed for radioguided surgery in prostate cancer, and our Nuclear Medicine service has successfully demonstrated the proof-of-concept of this new radiopharmaceutical. Previously, our service used [^68^Ga]Ga-PSMA-11 PET imaging for diagnosing and staging PCa patients. (da Cunha et al., 2018; Ringheim et al., 2020; Fuscaldi et al., 2021). Our clinical case report confirmed the value of [^99m^Tc]Tc-PSMA-I&S, obtained using this PSMA-I&S cold kit, in PCa patient. As shown in Figure 7, the small metastatic lymph node, detected as positive on [^68^Ga]Ga-PSMA-11 PET/CT study and further characterized on [^99m^Tc]Tc-PSMA-I&S SPECT/CT study, was successfully identified and resected with radioguided assisted technique.

## 5 Conclusion

The PSMA-I&S cold kit was radiolabeled with ^99m^Tc and validated through *in vitro* and *ex vivo* assays, resulting in a final product exhibiting a RCY of ∼90% and good radiochemical stability. The radiolabeling process, along with the quality control methods, can be completed in approximately 30–40 min, making them highly suitable for routine use in hospital Radiopharmacies and Nuclear Medicine services. Initial clinical applications have confirmed the potential of this radiopharmaceutical for radioguided surgery and SPECT imaging in patients with PCa.

## Data Availability

The raw data supporting the conclusion of this article will be made available by the authors, without undue reservation.
