# Corrigendum: Radiochemical and biological assessments of a PSMA-I&S cold kit for fast and inexpensive ^99m^Tc-labeling for SPECT imaging and radioguided surgery in prostate cancer

**DOI:** 10.3389/fchem.2024.1539768

**Published:** 2024-12-24

**Authors:** Leonardo Lima Fuscaldi, Danielle Vieira Sobral, Ana Claudia Durante, Fernanda Ferreira Mendonça, Ana Cláudia Camargo Miranda, Carla Salgueiro, Silvia Gomez de Castiglia, Lilian Yuri Itaya Yamaga, Marcelo Livorsi da Cunha, Luciana Malavolta, Marycel Figols de Barboza, Jorge Mejia

**Affiliations:** ^1^ Hospital Israelita Albert Einstein, Sao Paulo, Brazil; ^2^ Department of Physiological Sciences, Santa Casa de Sao Paulo School of Medical Sciences, Sao Paulo, Brazil; ^3^ Departamento de Química, Universidad Kennedy, Buenos Aires, Argentina; ^4^ Tecnonuclear-Eckert Ziegler, Buenos Aires, Argentina

**Keywords:** PSMA-I&S, technetium-99m, cold kits for radiopharmaceuticals, SPECT imaging, radioguided surgery, prostate cancer

In the published article, there was an error in **2 Materials and methods**, *2.10 Clinical Case*, paragraph 1. This work includes a SPECT scan of a human patient, which was said to have received approval from the institutional ethics committee. However, the project’s protocol number does not correspond to the indicated patient. This sentence previously stated:

“The study received approval from the institutional ethics committee (CAAE: 66235122.0.0000.0071).”

The corrected sentence appears below:

“The pilot study received approval from the institutional ethics committee and the patient signed the corresponding informed consent to use the SPECT images for research purposes.”

Consequently, there was also an error in the Ethics statement. The original statement was as follows:

“The animal study was approved by Ethics Committee on Animal Use of the Hospital Israelita Albert Einstein (protocol #4005/19). The study was conducted in accordance with the local legislation and institutional requirements. The human study was approved by the Ethics Committee of the Hospital Israelita Albert Einstein (CAAE: 66235122.0.0000.0071) and informed consent was obtained from all individual participants included in this study.”

The corrected statement appears below:

“The animal study was approved by Ethics Committee on Animal Use of the Hospital Israelita Albert Einstein (protocol #4005/19). The study was conducted in accordance with the local legislation and institutional requirements. The pilot human study was approved by the Ethics Committee of the Hospital Israelita Albert Einstein and informed consent was obtained from the participant included in this study.”

The authors apologize for these errors and state that this does not change the scientific conclusions of the article in any way. The original article has been updated.

